# Prenatal Care of Infants

**Published:** 1916-02

**Authors:** 


### Prenatal Care of Infants.

At a recent session of the Dallas Social Service School, Dr. Frank A. Pierce delivered an excellent address on the prenatal care of infants. He emphasized the fact that more attention should be given to the mothers before the birth of their children in order that children may have a fair start at birth. That suggests that the many Mothers' Clubs of the country would be doing a great work by inviting capable physicians to talk on the many health problems of the home and the community. So much sickness is unnecessary, and is the result of ignorance. The people are clamoring for enlightenment, and there are not many physicians who are so narrow that they will decline an invitation to tell how to conserve health.

